# CTRP-1 levels are related to insulin resistance in pregnancy and gestational diabetes mellitus

**DOI:** 10.1038/s41598-020-74413-1

**Published:** 2020-10-15

**Authors:** Carola Deischinger, Karoline Leitner, Sabina Baumgartner-Parzer, Dagmar Bancher-Todesca, Alexandra Kautzky-Willer, Jürgen Harreiter

**Affiliations:** 1 Department of Internal Medicine III, Clinical Division of Endocrinology and Metabolism, Gender Medicine Unit, Medical University of Vienna, Waehringer Guertel 18–20, 1090 Vienna, Austria; 2 Department of Obstetrics and Gynecology, Medical University of Vienna, Waehringer Guertel 18–20, 1090 Vienna, Austria

**Keywords:** Biomarkers, Endocrinology, Translational research, Reproductive disorders

## Abstract

Recent studies have shown higher levels of CTRP-1 (C1QTNF-related protein) in patients with type 2 diabetes compared to controls. We aimed at investigating CTRP-1 in gestational diabetes mellitus (GDM). CTRP-1 levels were investigated in 167 women (93 with normal glucose tolerance (NGT), 74 GDM) of a high-risk population for GDM. GDM was further divided into GDM subtypes depending on a predominant insulin sensitivity issue (GDM-IR) or secretion deficit (GDM-IS). Glucose tolerance was assessed with indices [Matsuda index, Stumvoll first phase index, insulin-secretion-sensitivity-index 2 (ISSI-2), area-under-the-curve (AUC) insulin, AUC glucose] derived from an oral glucose tolerance test (oGTT) performed at < 21 and 24–28 weeks of gestation. In pregnancy, CTRP-1 levels of GDM (76.86 ± 37.81 ng/ml) and NGT (82.2 ± 35.34 ng/ml; p = 0.104) were similar. However, GDM-IR women (65.18 ± 42.18 ng/ml) had significantly lower CTRP-1 levels compared to GDM-IS (85.10 ± 28.14 ng/ml; p = 0.009) and NGT (p = 0.006). CTRP-1 levels correlated negatively with weight, AUC insulin, Stumvoll first phase index, bioavailable estradiol and positively with HbA1c, Matsuda Index and ISSI-2. A multiple regression analysis revealed bioavailable estradiol (β = − 0.280, p = 0.008) and HbA1c (β = 0.238; p = 0.018) as the main variables associated with CTRP-1 in GDM. Postpartum, waist and hip measurements were predictive of CRTP-1 levels instead. CTRP-1 levels were higher postpartum than during pregnancy (91.92 ± 47.27 vs.82.44 ± 38.99 ng/ml; p = 0.013). CTRP-1 is related to insulin resistance in pregnancy and might be a metabolic biomarker for insulin resistance in GDM. CTRP-1 levels were significantly lower during pregnancy than postpartum, probably due to rising insulin resistance during pregnancy.

## Introduction

If the maternal pancreatic islets fail to cope with the increased demands in insulin production and secretion in pregnancy^1,2^, gestational diabetes mellitus (GDM) can develop. GDM, a form of hyperglycemia with its onset or first detection during pregnancy, has a prevalence of 2–6% in Europe^3^ and is associated with an increased risk for complications for both mother and child during pregnancy, childbirth and postpartum^4–7^. Women who suffer from GDM are found to have a nearly 3.5-fold elevated risk for developing prediabetes or type 2 diabetes mellitus postpartum^5^.

C1QTNF-related proteins (CTRPs) have been proposed as new possibilities for early detection and treatment in diabetes mellitus^25,26^. CTRP-1 is a member of the complement C1q tumor necrosis factor (C1QTNF) superfamily of proteins which includes well-known adipokines like adiponectin as well as the more recently identified C1QTNF-related proteins 1 to 15 (CTRP 1–15), all primarily expressed by adipose tissue^8–10^. In general, the C1QTNF superfamily is connected to inflammatory processes, autoimmunity, cell differentiation, apoptosis^11^ and has been related to sepsis^12^, preeclampsia^13^, incidence of major cardiovascular events^14^ and insulin-resistant obesity^11,15–18^, amongst others. CTRP-1, -2, -3, -9 and -12 have demonstrated to increase insulin sensitivity in previously conducted studies^9,10,19,20^ and protect against hyperglycemia by enhancing glycolysis and fatty acid oxidation^21^. Specifically, CTRP-1 has shown to improve insulin resistance by reducing the serine phosphorylation of IRS-1^10^. Accordingly, CTRP-1, -3 and -12 have displayed significantly different levels amongst patients with type 2 diabetes or prediabetes compared to NGT controls^10,22–24^.

Therefore, we aimed at investigating CTRP-1 in pregnancy and GDM. Because of the possible involvement of CTRP-1 in insulin resistance, CTRP-1 levels were further investigated in the context of insulin resistance and secretion to possibly establish differences between GDM subtypes.

## Results

### Baseline characteristics and glycemic profile

Baseline characteristics and details on the glycemic profile of all GDM subtypes as well as NGT women on the baseline visit (< 21st GW), GW 24–28 and postpartum are presented in Table 1A,B.

**Table 1:** Baseline characteristics and glycemic parameters of (A) oGTT at baseline (< 21st GW) and GW 24–28 and (B) postpartum oGTT and fetal parameters of all study participants, NGT and GDM by subtype (NGT, GDM-IR, GMD-IS, GDM-IR/IS).

	All	NGT	GDM	GDM-IS	GDM-IR	GDM-IR/IS
	Mean (± SD)	Mean (± SD)	Mean (± SD)	Mean (± SD)	Mean (± SD)	Mean (± SD)
**(A)**						
Maternal indices (< 21st GW)						
Number	167	92	75	30	31	14
Week of gestation	16 (± 3)	16 (± 3)	15 (± 3)	15 (± 4)	15 (± 2)	15 (± 4)
GDM in previous pregnancy	47.1%	37.8% *	57.8% *	64.29%	58.3%	41.6%
Birth weight > 4000 g in previous pregnancy	20.9%	13.9%	28.8%	26.7%	39.1%	15.4%
Weight (in kg)	80.6 (± 18.3)	79.8 (± 19.3)	81.7 (± 17.1)	73.2 (± 11.7) ^§§^	88.8 (± 18.5) ^§§^	84.3 (± 16.5)
Height (in cm)	164.91 (± 6.49)	166.08 (± 6.68) **	163.45 (± 5.96) **	162.91 (± 5.2)	163.45 (± 6.96)	165.3 (± 5.56)
BMI before pregnancy (in kg/m^2^)	29.61 (± 6.17)	28.86 (± 6.51) *	30.52 (± 5.62) *	27.68 (± 4.33) ^§§^	32.86 (± 5.94) ^§§^	31.44 (± 4.89)
Waist (in cm)	110.9 (± 11.3)	111.7 (± 7.4)	110.1 (± 14.8)	107.0 (± 0.0)	112.3 (± 15.9)	98.7 (± 4.7)
Hip (in cm)	121.6 (± 11.4)	121.7 (± 8.1)	121.5 (± 14.6)	119.0 (± 9.9)	124.7 (± 14.3) ^+^	104.3 (± 4.5) ^+^
Age (in years)	32 (± 5)	32 (± 5)	33 (± 5)	32 (± 5)	33 (± 5)	33 (± 4)
Systolic blood pressure (in mmHg)	116 (± 12)	115 (± 11) *	118 (± 13) *	114 (± 12)	120 (± 14)	122 (± 9)
Diastolic blood pressure (in mmHg)	70 (± 9)	68 (± 8) *	72 (± 9) *	69 (± 8)	74 (± 10)	74 (± 8)
Baseline visit (< 21st GW)						
Number	151	81	70	29	27	14
Matsuda Index	7.16 (± 4.95)	8.47 (± 5.4) **	5.73 (± 3.97) **	9.36 (± 3.36) ^§§§^°°	3.20 (± 2.43) ^§§§^	3.70 (± 1.42) °°
Stumvoll first phase index	1096.26 (± 699.81)	1161.43 (± 519.16)	1022.21 (± 858.78)	582.87 (± 252.80) ^§§§^	1650.42 (± 1044.84) ^§§§+++^	671.45 (± 396.77) ^+++^
ISSI-2	322.89 (± 140.88)	395.38 (± 127.39) ***	243.81 (± 109.26) ***	292.11 (± 115.13)	221.38 (± 92.88)	195.79 (± 97.19)
AUC insulin	60.83 (± 42.54)	53.25 (± 29.24) *	68.98 (± 52.28) *	37.73 (± 21.32) ^§§§^	103.45 (± 65.24) ^§§§++^	65.24 (± 18.98) ^++^
AUC glucose	127.00 (± 28.99)	109.10 (± 15.02) ***	146.24 (± 28.10) ***	145.55 (± 32.70)	140.91 (± 21.39)	157.46 (± 28.24)
CTRP-1 (in ng/ml)	84.37 (± 41.36)	85.57 (± 42.71)	82.99 (± 40.01)	89.99 (± 35.67)	70.90 (± 40.34)	91.79 (± 44.76)
HbA1c (in %)	5.2 (± 0.5)	5.1 (± 0.3) **	5.4 (± 0.6) **	5.4 (± 0.7)	5.3 (± 0.4)	5.4 (± 0.4)
Bioavailable estradiol (in ng/l)	612 (± 327)	582 (± 280)	645 (± 371)	545 (± 274)	769 (± 463)	614 (± 294)
Bioavailable testosterone (ng/ml)	0.05 (± 0.05)	0.04 (± 0.02) **	0.07 (± 0.06) **	0.05 (± 0.02)	0.08 (± 0.06)	0.06 (± 0.05)
GW 24–28						
Number	153	88	65	27	24	14
Matsuda Index	5.65 (± 4.15)	6.21 (± 4.40) *	3.95 (± 2.65) *	6.65 (± 1.98) ^§^	1.77 (± 0.86) ^§^	2.93 (± 0.47)
Stumvoll first phase index	1248.00 (± 731.15)	1287.37 (± 599.77)	1125.18 (± 1047.43)	524.52 (± 168.70) ^§§§^	1924.14 (± 1299.90) ^§§§++^	728.59 (± 149.37) ^++^
ISSI-2	320.85 (± 138.61)	355.22 (± 135.04) ***	210.59 (± 81.43) ***	263.69 (± 91.23)	178.03 (± 50.74)	159.25 (± 41.30)
AUC insulin	75.92 (± 63.17)	66.44 (± 32.14)	105.12 (± 111.12)	51.06 (± 12.52) ^§§§^	173.30 (± 153.78) ^§§§+^	76.88 (± 24.36) ^+^
AUC glucose	125.96 (± 22.79)	115.63 (± 12.76) ***	157.78 (± 16.55) ***	159.84 (± 11.04)	158.21 (± 18.92)	152.78 (± 22.84)
CTRP-1 (in ng/ml)	79.93 (± 36.38)	82.2 (± 35.34) ^^	76.86 (± 37.81)	85.10 (± 28.14) ^§§^	65.79 (± 42.18) ^^ ^§§^	79.95 (± 43.90)
HbA1c (in %)	5.0 (± 0.4)	4.9 (± 0.4) **	5.2 (± 0.4) **	5.2 (± 0.5)	5.1 (± 0.3)	5.2 (± 0.4)
Bioavailable estradiol (in ng/l)	1377 (± 519)	1366 (± 499)	1392 (± 547)	1239 (± 401)	1583 (± 642)	1361 (± 554)
Bioavailable testosterone (ng/ml)	0.04 (± 0.03)	0.04 (± 0.03)	0.05 (± 0.04)	0.04 (± 0.02)	0.05 (± 0.04)	0.06 (± 0.06)
**(B)**						
Postpartum visit						
Number	145	79	66	29	23	14
Matsuda Index	8.93 (± 8.02)	10.39 (± 10.23)	8.13 (± 6.52)	10.80 (± 7.46)	4.94 (± 3.86)	5.57 (± 3.53)
Stumvoll first phase index	1244.48 (± 2890.23)	1919.88 (± 4766.75)	872.32 (± 588.64)	706.37 (± 513.95)	1220.68 (± 667.36)	837.78 (± 516.58)
ISSI-2	286.98 (± 139.59)	345.09 (± 149.37) **	255.50 (± 124.56) **	268.98 (± 130.11)	233.81 (± 121.16)	249.94 (± 123.03)
AUC insulin	48.51 (± 32.48)	43.82 (± 31.56)	51.05 (± 33.02)	40.17 (± 28.83) ^§^	69.48 (± 43.53) ^§^	55.65 (± 17.87)
AUC glucose	125.54 (± 29.51)	105.6 (± 19.08) ***	136.35 (± 28.63) ***	133.76 (± 27.63)	134.94 (± 22.90)	143.77 (± 36.92)
CTRP-1 (in ng/ml)	91.92 (± 47.27)	88.93 (± 47.36)	96.26 (± 47.24)	110.82 (± 33.11) ^§§^	72.27 (± 51.73) ^§§^	101.91 (± 52.58)
HbA1c (in %)	5.4 (± 0.8)	5.30 (± 0.4)	5.50 (± 1.0)	5.6 (± 1.5)	5.4 (± 0.3)	5.6 (± 0.5)
Bioavailable estradiol (in ng/l)	38 (± 52)	36 (± 47)	41 (± 57)	57 (± 80)	34 (± 31)	36 (± 23)
Bioavailable testosterone (ng/ml)	0.05 (± 0.05)	0.08 (± 0.06)	0.11(± 0.07)	0.11 (± 0.07)	0.10 (± 0.07)	0.13 (± 0.08)
Fetal parameters						
Number	167	92	75	30	31	14
Birth weight (in grams)	3388 (± 588)	3413 (± 507)	3358 (± 678)	3263 (± 748)	3520 (± 568)	3185 (± 731)
Birth length (in cm)	51.1 (± 3.2)	51.4 (± 3.0)	50.7 (± 3.4)	50.2 (± 3.8)	51.5 (± 2.6)	49.6 (± 3.9)
Head circumference (in cm)	34.8 (± 3.4)	35.3 (± 3.0) *	34.2 (± 4.2) *	33.7 (± 6.3)	34.7 (± 2.8)	33.8 (± 2.1)
Abdominal circumference (in cm)	31.0 (± 2.9)	31.0 (± 2.5)	31.0 (± 3.4)	24.5 (± 5.7)	31.8 (± 3.2)	28.7(± 0.9)
Gestation week at birth (in weeks)	39 (± 2)	40 (± 1)	39 (± 2)	39 (± 2)	39 (± 1)	39 (± 3)

Continuous variables were summarized by mean ± standard deviation (SD) and categorical variables by counts and percentages. To assess differences between NGT, GDM and GDM subgroups, an independent samples T-test and a One-Way ANOVA with Tukey correction for multiple testing was performed.

NGT vs GDM: *the significance level is p < 0.05; **the significance level is p < 0.01; ***the significance level is p < 0.001.

NGT vs GDM-IR: ^^^the significance level is p < 0.05; ^^^^the significance level is p < 0.01; ^^^^^the significance level is p < 0.001.

GDM-IS vs GDM-IR: ^§^the significance level is p < 0.05; ^§§^the significance level is p < 0.01; ^§§§^the significance level is p < 0.001.

GDM-IR vs. GDM-IR/IS: ^+^the significance level is p < 0.05; ^++^the significance level is p < 0.01; ^+++^the significance level is p < 0.001.

GDM-IS vs. GDM-IR/IS: ^°^the significance level is p < 0.05; ^°°^the significance level is p < 0.01; ^°°°^the significance level is p < 0.001.

There were significant differences in glycemic parameters between NGT and GDM as well as between the GDM groups. Women with NGT showed better glycemic control and a higher percentage of patients with GDM had had GDM in a previous pregnancy than women with NGT. Within the GDM-groups, GDM-IS and GDM-IR displayed the most striking differences. BMI before pregnancy (p = 0.003) and weight (p = 0.004) were lower in the GDM-IS group. Concerning glycemic control parameters, the Matsuda index (p < 0.001) was lower and the Stumvoll first phase index (p < 0.001) higher in women of the GDM-IR group. AUC insulin (p < 0.001) was by far the highest in GDM-IR women. There were no significant differences in fetal parameters except for greater head circumference in neonates from women with NGT.

### Differences in CTRP-1 levels between groups during pregnancy and postpartum

At GW 24 to 28, CTRP-1 levels were significantly lower in the GDM-IR group than GDM-IS (p = 0.009) and women with NGT (p = 0.006). CTRP-1 levels were marginally higher in NGT compared to all women with GDM (p = 0.104), however, this difference was not statistically significant. Postpartum, CTRP-1 levels were also lower in the GDM-IR group than in GDM-IS (p = 0.002). CTRP-1 levels increased postpartum compared to during pregnancy (p = 0.013).

### Correlation of CTRP-1 with covariates in pregnancy

There was no correlation between the week of gestation and CTRP-1 levels, showing no dynamic in CTRP-1 levels throughout pregnancy. During pregnancy, there was a significant positive correlation between CTRP-1 and the Matsuda Index, ISSI-2, HbA1c and a negative correlation with AUC insulin, Stumvoll first phase index, weight and bioavailable estradiol. When subjected to multiple regression analysis, HbA1c and ISSI-2 were predictive of serum CTRP1 levels for all women in pregnancy (see Table 2).

**Table 2:** Pearson’s correlation analysis and multiple regression analysis of CTRP-1 during pregnancy.

Pregnancy	All (in pregnancy)				GDM (in pregnancy)				NGT (in pregnancy)			
	Simple		Multiple regression		Simple		Multiple regression		Simple		Multiple regression	
	R_P_	P	β	P	R_P_	P	β	P	R_P_	P	β	P
Week of gestation	0.000	0.996			− 0.044	0.629			0.038	0.626		
Weight	**− 0.173**	**0.003**	− 0.065	0.393	− 0.139	0.122			**− 0.199**	**0.011**	− 0.038	0.773
Height	0.084	0.154			0.135	0.135			0.026	0.743		
Waist	0.056	0.572			− 0.039	0.816			0.059	0.637		
Hip	0.028	0.781			0.143	0.391			− 0.063	0.614		
Age	0.009	0.880			0.078	0.390			− 0.031	0.696		
RR systolic	0.015	0.807			− 0.002	0.985			0.053	0.504		
RR diastolic	− 0.057	0.332			− 0.077	0.394			− 0.011	0.887		
Matsuda Index	**0.269**	** < 0.001**	− 0.048	0.672	**0.353**	**0.001**	0.136	0.415	**0.210**	**0.011**	0.125	0.580
Stumvoll first phase index	**− 0.257**	** < 0.001**	− 0.145	0.156	**− 0.241**	**0.028**	− 0.105	0.442	**− 0.335**	** < 0.001**	− 0.281	0.212
ISSI-2	**0.218**	**0.001**	**0.258**	**0.003**	**0.256**	**0.021**	0.146	0.291	0.159	0.056	− 0.041	0.813
AUC insulin	**− 0.213**	**0.001**	− 0.106	0.322	**− 0.226**	**0.040**	0.075	0.691	**− 0.181**	**0.029**	0.118	0.602
AUC glucose	0.009	0.894			0.093	0.406			0.117	0.160		
HbA1c	**0.160**	**0.006**	**0.156**	**0.027**	**0.259**	**0.004**	**0.238**	**0.018**	0.119	0.130		
Adiponectin	0.098	0.182			0.092	0.384			0.070	0.496		
usCRP	− 0.079	0.184			0.019	0.836			**− 0.168**	**0.032**	− 0.034	0.802
Estradiol	− 0.048	0.419			− 0.080	0.377			− 0.027	0.736		
Bioavailable estradiol	**− 0.122**	**0.039**	− 0.045	0.515	**− 0.240**	**0.007**	**− 0.280**	**0.008**	− 0.015	0.849		
Testosterone	− 0.083	0.316			0.131	0.315			**− 0.279**	**0.009**	**− 0.255**	**0.042**
Bioavailable testosterone	− 0.058	0.488			0.153	0.243			− 0.210	0.057	− 0.533	0.063

All variables with p < 0.1 were included in the multiple regression analysis to test the joint effect of these parameters on CTRP-1. Variables with a VIF > 3.5 in the multicollinearity diagnostic were excluded from the analysis.

Bold indicates the significance level is p < 0.05.

**Figure 1:**
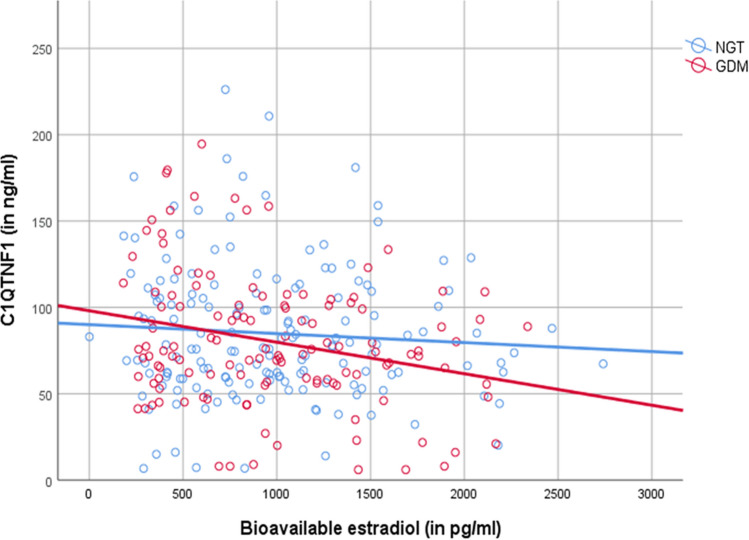
Grouped scatter dot plot with fit line comparing the correlation between bioavailable estradiol in women with NGT and GDM during pregnancy.

In women with GDM, CTRP-1 correlated negatively with estradiol and bioavailable estradiol during pregnancy (see also Fig. 1). Here, a multiple regression analysis revealed bioavailable estradiol and HbA1c as the main variables predictive of CTRP-1 levels. Contrastingly, as illustrated in Fig. 1, no correlation of estradiol and CTRP-1 could be found in NGT.

In women with NGT, CTRP correlated negatively with weight, Stumvoll first phase index, AUC insulin, testosterone levels, usCRP and positively with Matsuda index and ISSI-2. When subjected to multiple regression analysis, testosterone was predictive of serum CTRP1 levels for women with NGT in pregnancy (see Table 2).

### Correlation of CTRP-1 with covariates postpartum

Postpartum, sex hormones such as bioavailable estradiol seized to correlate significantly with CTRP-1 (see Table 3). Instead, AUC glucose correlated positively; weight, diastolic blood pressure, waist and hip measurements negatively with CTRP-1. In women who had had GDM in their pregnancy, CTRP-1 correlated with Stumvoll first phase levels. Here, the conducted multiple regression analysis identified Stumvoll first phase levels as predictive of CTRP-1. For women with NGT in their pregnancy, age and hip measurements were predictive for CTRP-1 levels (see Table 3). In all women together, waist and hip measurements were predictive of CTRP-1 levels.

**Table 3:** Pearson’s correlation analysis and multiple regression analysis of CTRP1 postpartum.

Postpartum	All (postpartum) N = 145				GDM (Postpartum) N = 66				NGT (postpartum) N = 79			
	Simple		Multiple regression		Simple		Multiple regression		Simple		Multiple regression	
	R_P_	P	β	P	R_P_	P	β	P	R_P_	P	β	P
Weight	**− 0.301**	** < 0.001**	− 0.048	0.910	**− 0.331**	**0.011**	0.258	0.507	**− 0.281**	**0.014**	− 0.127	0.716
Height	− 0.025	0.763			**0.759**	** < 0.001**	0.214	0.140	0.053	0.664		
Waist	**− 0.208**	**0.015**	**0.517**	**0.015**	− 0.169	0.201			**− 0.250**	**0.030**	0.172	0.382
Hip	**− 0.303**	** < 0.001**	**− 0.473**	**0.029**	**− 0.277**	**0.034**	− 0.365	0.351	**− 0.323**	**0.005**	**− 0.431**	**0.032**
Age	− 0.153	0.066	− 0.065	0.615	0.111	0.377	0.291	0.064	**− 0.374**	**0.001**	**− 0.419**	** < 0.001**
RR systolic	− 0.100	0.251			− 0.137	0.306			− 0.061	0.603		
RR diastolic	**− 0.203**	**0.018**	0.115	0.372	**− 0.280**	**0.033**	− 0.031	0.818	− 0.132	0.256		
HbA1c	0.082	0.326			0.116	0.356			0.022	0.849		
Matsuda Index	0.168	0.152			0.285	0.050	− 0.042	0.806	0.112	0.586		
Stumvoll first phase index	0.017	0.887			**− 0.440**	**0.002**	**− 0.456**	**0.009**	0.134	0.504		
ISSI-2	− 0.002	0.085	0.231	0.131	0.229	0.117			− 0.162	0.429		
AUC insulin	− 0.136	0.249			**− 0.360**	**0.012**	− 0.089	0.772	− 0.128	0.534		
AUC glucose	**0.232**	**0.047**	0.271	0.076	0.151	0.306			0.302	0.134		
Adiponectin	0.009	0.932			− 0.044	0.773			0.068	0.658		
Estradiol	− 0.081	0.335			− 0.031	0.809			− 0.107	0.350		
Bioavailable estradiol	− 0.110	0.195			− 0.047	0.718			− 0.117	0.120		

All variables with p < 0.1 were included in the multiple regression analysis to test the joint effect of these parameters on CTRP1. Variables with a VIF > 3.5 in the multicollinearity diagnostic were excluded from the analysis.

Bold indicates the significance level is p < 0.05.

## Discussion

Although studies on patients with type 2 diabetes showed higher CTRP-1 levels in diabetes mellitus patients compared to non-diabetic controls^25^, there was no significant difference in CTRP-1 levels between women with GDM and those with NGT in our pregnant cohort except for the subgroup GDM-IR. However, CTRP-1 has been related to insulin sensitivity in previous studies^12,25^, which is corroborated by our results. CTRP-1 levels were significantly lower in women with GDM-IR than in women with GDM-IS and NGT pointing towards CTRP-1 being a parameter associated with insulin resistance in GDM. During pregnancy, CTRP-1 correlated significantly with parameters associated with insulin sensitivity and sex hormones. Postpartum, CTRP-1 ceased to be influenced by glycemic indices and sex hormones and correlated with weight, waist and hip measurements instead, which is in line with studies in non-pregnant cohorts showing a relationship between obesity and CTRPs and CTRP-1 predominantly being expressed by adipose tissue^8,25,27^.

Data on CTRP-1 and pregnancy is limited to one study investigating CTRP-1 in a cohort of women with preeclampsia where a positive correlation with blood pressure was found^13^. In our cohort, there was no connection of CTRP-1 with blood pressure levels during pregnancy, however, a negative correlation with diastolic blood pressure in the postpartum visit. However, we were able to demonstrate a connection between CTRP-1, glucose metabolism and insulin resistance in pregnancy and GDM similar to previously published studies including non-pregnant individuals^25^. In our results, CTRP-1 showed a positive correlation with the Matsuda Index and ISSI-2 as well as a negative correlation with AUC Insulin. As the Matsuda Index is an estimate for insulin resistance, higher CTRP-1 levels might be associated with better insulin sensitivity in pregnancy. In general, the increase in insulin resistance during pregnancy is a physiological process needed for the glucose supply of the fetus^28^. From the second trimester onwards, insulin resistance physiologically increases via a reduced insulin-stimulated muscular glucose transport^29,30^. This multifactorial process involves a reduced ability of insulin to phosphorylate tyrosine residues of IRS-1, which then leads to lower levels of glucose transporter 4 (GLUT-4) translocation^30,31^. Insulin-stimulated glucose transport was even more greatly impaired in women with GDM who had lower levels of IRS-1 compared to their BMI-matched controls with NGT^32^. This might stem from either degradation of IRS-1 resulting from a defective tyrosine IRS-1 phosphorylation or the activation of the mTor-p70S6 pathway which both lead to serine phosphorylation and, consequently, IRS-1 degradation and lower GLUT-4 translocation^30^. CTRP-1 has shown to improve insulin resistance by reducing the serine phosphorylation of IRS-1^10^. A study demonstrated a correlation of CTRP-1 with glucose metabolism and insulin resistance by showing that GLUT-4 translocation and AMP-activated protein kinase (AMPK) activation were significantly lower in the skeletal muscle of CTRP-1 knock out mice^27^. Correspondingly, CTRP-1 levels were significantly lower in women with GDM-IR than in women with GDM-IS and NGT in our analysis.

However, studies on patients with type 2 diabetes showed higher CTRP-1 levels in this population than non-diabetic controls^25^, whereas we observed the opposite effect, namely, significantly lower CTRP-1 levels in the GDM-IR group compared to GDM-IS or NGT in our study. Along these lines, CTRP-1 correlated negatively with BMI in our analysis, but positively in previous studies^12,25^. The reverse results on CTRP-1 levels in pregnancy could be explained by hormonal changes in pregnancy, more specifically, the physiological increase in estrogen levels during gestation. A multiple regression analysis, which included correlating glucose tolerance indices, weight, height and estradiol, revealed bioavailable estradiol and HbA1c as the main variables linked to CTRP-1 levels in women with GDM. In NGT, testosterone levels were associated with CTRP-1 in the multiple regression analysis. No data on CTRP-1 and testosterone are currently available. However, a connection between another member of the C1qTNF superfamily, CTRP-3, and testosterone could be found in previously published studies where CTRP-3 stimulated testosterone production^33^. Furthermore, CTRP-3 and CTRP-6 levels were lower in men than women^8,34^. In the present study, low CTRP-1 levels were associated with increased insulin resistance. Considering the connection between insulin resistance and elevated testosterone in pregnant women^35^, it seems fitting that testosterone levels correlated negatively with CTRP-1 in the NGT group. Postpartum, the negative correlation of CTRP-1 with estradiol and testosterone ceased to be significant and waist and hip measurements were predictive of CTRP-1 levels instead, which might indicate sex hormones as strong influencing factors during pregnancy. Correspondingly, we found that CTRP-1 levels increased postpartum again. According to previously published studies, the increase in insulin resistance during pregnancy can partly be attributed to changing sex hormone levels such as human placenta lactogen (hPL), human placenta growth hormone (hPGH) or estradiol^36^. In a study on mice, Palazzo et al. demonstrated that an oophorectomy increased, a 17β-estradiol treatment decreased insulin sensitivity via GLUT4 gene expression in skeletal muscle tissue^29^. Thus, a state of hyperestrogenism such as in pregnancy and GDM might lead to a reduced GLUT-4 translocation and, consequently, impaired insulin-stimulated muscular glucose transport^29^. Therefore, we hypothesize that sex hormones such as estradiol might lower CTRP-1 to facilitate insulin resistance in pregnancy.

A strength of our analysis is the high number of women with GDM, which allowed us to differentiate between different GDM subtypes. Our study population had an especially high proportion of women with an elevated risk for GDM due to our center being a specialist facility. Therefore, not only the women with GDM but also a large proportion of women with NGT had risk factors for GDM such as an overweight/obese BMI, which we corrected for in the multiple regression analysis^8^. Furthermore, in some women, specific values of the oGTT are missing, which then were pairwise excluded (< 10% of the population). Strength and limitation at the same time is that CTRP-1, to the best of our knowledge, has only been investigated in a pregnant population once before^13^. Although CTRP-1 is, therefore, a potential new area of investigation, the suitability of CTRP-1 ELISA kits for pregnancy is unclear.

With lower levels in women with GDM-IR than NGT or GDM-IS, CTRP-1 levels might be related to insulin resistance in pregnancy and gestational diabetes mellitus. In pregnancy, increasing peripheral insulin resistance and central leptin resistance diverts glucose to the placenta and, thus, the fetus^1^. Correspondingly, CTRP-1 levels were significantly higher postpartum compared to during pregnancy. However, whether the downregulation of CTRP-1 in pregnancy is facilitating or rather a consequence of the rise in insulin resistance remains unknown and requires further research on CTRP-1, its possible roles in pregnancy and the connection of CTRP-1, insulin resistance and sex hormones. Nevertheless, we were able to demonstrate that the previously published connection between CTRP-1, glucose metabolism and insulin resistance in non-pregnant individuals^25^ can be replicated in pregnancy and GDM.

## Materials and methods

### Study participants and design

This pilot study included 167 pregnant women (predominantly Caucasian) participating in two prospective longitudinal studies conducted at the Medical University of Vienna between 2010 and 2014. The studies were approved by the local ethics committee (Ethics Committee of the Medical University of Vienna, EK Nr. 2022/2012 and 771/2008) and performed in accordance with the Declaration of Helsinki. All subjects gave written informed consent for participation in the study^37^. Inclusion criteria were a singleton pregnancy and age ≥ 18 years. Exclusion criteria included pre-conceptional diabetes, pre-existing, chronic and/or infectious diseases, significant psychiatric disorders or inability to follow instructions related to the studies due to language difficulties. All study subjects were monitored during their pregnancy following the national guidelines^38^. As a center taking care of higher risk pregnancies, a high number of cases with GDM is represented in our cohort. An oral glucose tolerance test (OGTT) was performed at the baseline visit (< 21st GW), at visit 2 (24th–28th GW) and postpartum with blood samples taken at baseline, 30, 60, 90 and 120 min for the measurement of glucose and insulin. GDM was assessed according to IADPSG/WHO 2013 guidelines with either fasting plasma glucose between 92 and 125 mg/dl, 1-h plasma glucose > 180 mg/dl or 2-h plasma glucose between 153 and 199 mg/dl^39^. If the glucose tolerance test at < 21st GW showed GDM, no further OGTT was performed during pregnancy. All women with GDM were treated according to national guidelines at our facility^38^. Systolic and diastolic blood pressure were measured on the left arm with an OMRON 705 device. Patients were in resting position for at least two to three minutes before testing. An average of two measurements taken one min apart was recorded. Weight was measured to the nearest 0.1 kg on calibrated electronic scales (SECA 877/888) wearing no shoes and light clothes. Waist circumference was measured twice at the midpoint between the lower border of the rib cage and the iliac crest and hip circumference at the widest portion of the buttocks. An average of the two measurements was recorded.

### Assays

All samples were analyzed in our ISO 9001 certified central laboratory at the General Hospital in Vienna (AKH Wien). Methods are available under the homepage of the institute of laboratory medicine, www.kilm.at. CTRP-1 in serum was measured with the human ELISA kit SEK170Hu provided by the company Cloud Clone. The detection range of this kit is 1.56–100 ng/ml with an inter-assay CV of < 10% and an intra-assay CV of < 12%.

### Calculation of GDM subtypes according to insulin secretion and sensitivity indices

In order to investigate the link between CTRP-1 and glucose control in GDM, we used the distributions of insulin sensitivity and secretion in women with normal glucose tolerance (NGT) from the OGTT at < 21st GW and 24th–28th GW to define GDM subtypes. The approach is similar to the previously published one by Powe et al., who considered women with GDM to have a predominant insulin secretion or sensitivity defect if the Matsuda Index (used to assess insulin sensitivity) and Stumvoll first phase (for insulin secretion) were below the 25th percentile of the NGT values, respectively^40^. Consequently, women with GDM were divided into three groups, GDM with a predominant insulin secretion defect (GDM-IS), GDM with a predominant insulin resistance defect (GDM-IR) or GDM with a mixed defect (GDM-IR/IS). Women who were diagnosed with GDM at < 21st GW were assigned to a GDM group depending on the NGT cut-off values determined at the baseline visit. Accordingly, for women who were diagnosed at visit 2, the NGT values of GW 24–28 were used. For the GDM-IS group, this resulted in Stumvoll's first phase index cut-off values of < 902.53 (if diagnosed at baseline visit) and < 784.67 (if diagnosed at GW24-28). For the GDM-IR group, the Matsuda index ought to be < 4.33 (if diagnosed at baseline visit) and < 3.57 (if diagnosed at GW24-28). If both criteria are met, women were allocated to the GDM-IR/IS group. AUC insulin and AUC glucose were calculated with the trapezoidal method. The Matsuda Index is an estimate of peripheral and hepatic insulin sensitivity (liver, muscle and adipose tissue) and computed according to Matsuda et al.^41^. Stumvoll first phase insulin secretion was calculated as 1.283 + 1.829 × Insulin 30 min − 138.7 × Glucose 30 min + 3.772 × Insulin 0 min for estimated first phase beta-cell function^42^. To improve the assessment of beta-cell reserve, ISSI-2 (insulin secretion sensitivity index), the product of the Matsuda Index and the ratio of the area-under-the-insulin curve to the area-under-the-glucose curve, was used^43^.

### Statistical analysis

We compared CTRP-1 levels, baseline characteristics and pregnancy outcomes across GDM subtypes and the NGT group. Missing records were deleted pairwise. A descriptive data analysis was performed for all parameters where continuous variables were summarized by mean ± SD and categorical variables by counts and percentages. By visual assessment of histograms and calculation of skewness using the Kolmogorov–Smirnov test, normal distribution was determined. Nonparametrically distributed parameters were log-transformed. A One-Way Anova was performed with CTRP-1 as a dependent variable across GDM subgroups during all pregnancy visits. In the post hoc analysis, multiple testing was adjusted with Tukey correction. Correlation analysis was performed using Pearson's correlation. Parameters with p < 0.1 were entered in multiple regression analysis for detecting independent associations with CTRP-1 levels. Multicollinearity was tested in all linear regression models. Only parameters with a variance inflation factor below 3.5 were included in the multiple regression analysis. All entered variables were checked for normality with normal probability plots and homoscedasticity using scatter dot plots. Statistical analysis was performed using SPSS 25.0 (SPSS Inc, Chicago, USA). A two-sided p value < 0.05 was considered statistically significant.

## Abbreviations

AMPK: AMP-activated protein kinase
C1QTNF: C1q tumor necrosis factor
CTRP-1: C1QTNF-related protein
GDM: Gestational diabetes mellitus
GDM-IR: GDM with a predominant insulin resistance defect
GDM-IR/IS: GDM with a mixed defect
GDM-IS: GDM with a predominant insulin secretion defect
GLUT-4: Glucose transporter 4
hPGH: Human placenta growth hormone
hPL: Human placenta lactogen
IRS-1: Insulin receptor substrate 1
NGT: Normal glucose tolerance
OGTT: Oral glucose tolerance test
